# Genetic engineering for enhanced production of a novel alkaline protease BSP-1 in *Bacillus amyloliquefaciens*

**DOI:** 10.3389/fbioe.2022.977215

**Published:** 2022-08-30

**Authors:** Cong Jiang, Changwen Ye, Yongfeng Liu, Kuo Huang, Xuedeng Jiang, Dian Zou, Lu Li, Wenyuan Han, Xuetuan Wei

**Affiliations:** ^1^ State Key Laboratory of Agricultural Microbiology, Hubei Hongshan Laboratory, Huazhong Agricultural University, Wuhan, China; ^2^ Zhengzhou Tobacco Research Institute of China National Tobacco Corporation, Zhengzhou, China; ^3^ GeneMind Biosciences Company Limited, Shenzhen, China; ^4^ Sericultural & Argi-Food Research Institute, Guangdong Academy of Agricultural Sciences/Key Laboratory of Functional Foods, Ministry of Agriculture and Rural Affairs/Guangdong Key Laboratory of Agricultural Products Processing, Guangzhou, China

**Keywords:** alkaline protease, *bacillus amyloliquefaciens*, promoter screening, recombinant expression, fermentation optimization

## Abstract

Alkaline protease has been widely applied in food, medicine, environmental protection and other industrial fields. However, the current activity and yield of alkaline protease cannot meet the demand. Therefore, it is important to identify new alkaline proteases with high activity. In this study, we cloned a potential alkaline protease gene *bsp-1* from a *Bacillus subtilis* strain isolated in our laboratory*.* BSP-1 shows the highest sequence similarity to subtilisin NAT (S51909) from *B. subtilis* natto*.* Then, we expressed BSP-1 in *Bacillus amyloliquefaciens* BAX-9 and analyzed the protein expression level under a collection of promoters. The results show that the P43 promoter resulted in the highest transcription level, protein level and enzyme activity. Finally, we obtained a maximum activity of 524.12 U/mL using the P43 promoter after fermentation medium optimization. In conclusion, this study identified an alkaline protease gene *bsp-1* from *B. subtilis* and provided a new method for high-efficiency alkaline protease expression in *B. amyloliquefaciens*.

## 1 Introduction

Alkaline protease is found in all living organisms (Gupta et al., 2002). Alkaline protease has a lot of functions and it is widely applied in washing, food, textile, leather, pharmaceutical and other industries (Contesini et al., 2018; Harish and Uppuluri, 2018; Pathak and Rathod, 2018; Tavano et al., 2018; Jahangirian et al., 2019; Sharma et al., 2019; Burchacka et al., 2022). However, in recent years, the yield of alkaline protease fail to satisfy the industrial demand, causing the shortage problem of large-scale industrial enzymes (Barzkar, 2020). Therefore, developing efficient alkaline protease has great industrial prospects.

Many strains produce alkaline protease, *Bacillus* genus are the main producer of the enzyme in industrial production (Cai et al., 2019). *Bacillus* have high secretory capacity and become the main producer of the enzyme in industrial production (Westers et al., 2004). However, the yield and activity of alkaline protease in *Bacillus* is usually low (Zhang et al., 2020; Ouyang et al., 2021; Zhang et al., 2021; Espoui et al., 2022). To achieve high-efficiency expression of target proteins, researchers had reformed expression elements, such as signal peptides, transcription factors, and molecular chaperones (Liu et al., 2019; Suberu et al., 2019; Gong et al., 2020; Neef et al., 2021; Su et al., 2021). For instance, Gang et al. constructed a signal peptide library in *Bacillus subtilis*, and screened out the amylase activity up to 5086 U/mL (Fu et al., 2018). In addition, by combining the transcriptional activator Spo0A with the regulatory region of *Bacillus licheniformis*, Zhou et al. successfully achieved 1.46-fold increase in the yield of alkaline protease AprE (Zhou et al., 2020). In addition, since promoter is one of the most critical factors in gene expression regulation (Kumar and Bansal, 2018; Brázda et al., 2021; Cazier and Blazeck, 2021; Jensen and Galburt, 2021), its engineering also serves as an important strategy to increase microbial gene expression and metabolites production. Su et al. substituted the wild-type −10 box and −35 box of *aprN* promoter, resulting in significantly higher nattokinase production than those previously reported in *B. subtilis* (Wu et al., 2011). Liao et al. (2018) optimized the ribosome-binding site to improve β-Gal activity in *B. amyloliquefaciens*. Together, genetic manipulation of the above elements has been widely applied in the production of industrial enzymes.

*B. subtilis* has significant attributes, such as fast growth and development, strong metabolic capacity and rich products, *B. subtilis* has been the preferred organisms for industrial production of a variety of products (Gu et al., 2018)*. B. amyloliquefaciens* could secrete recombinant proteins using a variety of signal peptides (Cui et al., 2018), thus it had been an efficient platform for producing various proteases (Uyar and Baysal, 2008; Matkawala et al., 2021; Meng et al., 2021). In this study, we identified an alkaline protease gene *bsp-1* from *B. subtilis*, and then expressed it in *B. amyloliquefaciens* BAX-9. Subsequently, we further analyzed the expression level under different promoters. Finally, the highest expression level of alkaline protease was obtained by using the promotor P43 combined with fermentation optimization.

## 2 Materials and methods

### 2.1 Chemicals

In this study, the *TransStartFastPfu* DNA polymerase was purchased from TransGen Biotech Co., Ltd. (Beijing, China). Restriction enzymes, dNTPs, T4 ligase and RNase were provided by Takara Biotechnology Co., Ltd. (Dalian, China). We purchased the Total RNA Isolation Kit and PrimeScript RT Master Mix Kit from Vazyme Biotech Co.,Ltd. (Nanjing, China). Standard alkaline protease was purchased from Sigma-Aldrich Co., LLC (St.Louis, United States). All the other chemicals were obtained from Sinopharm Chemical Reagent Co., Ltd. (Shanghai, China).

### 2.2 Recombinant expression of *bsp-1* gene

To express BSP-1 in *B. amyloliquefaciens* BAX-9, we constructed the recombinant strains following the procedure reported in our previous study (Zou et al., 2020). All strains and plasmids involved were listed in Tables 1, 2, listed all designed primers. For example, a pair of primers were designed to amplify the *bsp-1* gene from *B. subtilis* DNA, Ppqq promoter from *B. amyloliquefaciens* and TamyL terminator from *B. licheniformis* WX-02 respectively, and then Ppqq promoter, TamyL terminator and *bsp-1* gene were fused by Splicing with Overlap Extension PCR (SOE-PCR). The fused fragment and the pHY-300PLK plasmid were digested by restriction enzymes of *BamH*I and *Xba*I, and then they were ligated to obtain the expression plasmid pHY-Ppqq/BSP-1. The expression plasmid pHY-Ppqq*/*BSP-1 was electroporated into BAX-9 to obtain the recombinant strain. In this study, the remaining recombinant strains were constructed using the same procedure.

**TABLE 1:** Strains and plasmids used in this study.

Strains	Characteristics	Source
BAX-9	*B. amyloliquefaciens* HZ-12 deficient in *epr, nprE, aprE, aprX, mpr, bpf, vpr, htrB, yktc1*	Stored in lab
BAX-9/pHY-300	BAX-9 harboring the plasmid pHY300PLK	Stored in lab
BAX-9/pHY-Psra/BSP-1	BAX-9 harboring the plasmid pHY-Psra/BSP-1	This study
BAX-9/pHY-Psrs/BSP-1	BAX-9 harboring the plasmid pHY-Psrs/BSP-1	This study
BAX-9/pHY-PrnpB/BSP-1	BAX-9 harboring the plasmid pHY-PrnpB/BSP-1	This study
BAX-9/pHY-Pffs/BSP-1	BAX-9 harboring the plasmid pHY-Pffs/BSP-1	This study
BAX-9/pHY-Phyp/BSP-1	BAX-9 harboring the plasmid pHY-Phyp/BSP-1	This study
BAX-9/pHY-Ppqq/BSP-1	BAX-9 harboring the plasmid pHY-Ppqq/BSP-1	This study
BAX-9/pHY-Pscp/BSP-1	BAX-9 harboring the plasmid pHY-Pscp/BSP-1	This study
BAX-9/pHY-Pcsp/BSP-1	BAX-9 harboring the plasmid pHY-Pcsp/BSP-1	This study
BAX-9/pHY-P43/BSP-1	BAX-9 harboring the plasmid pHY-P43/BSP-1	This study
BAX-9/pHY-PtrnQ/BSP-1	BAX-9 harboring the plasmid pHY-PtrnQ/BSP-1	This study
BAX-9/pHY-Psrf/BSP-1	BAX-9 harboring the plasmid pHY-Psrf/BSP-1	This study
BAX-9/pHY-Pitu/BSP-1	BAX-9 harboring the plasmid pHY-Pitu/BSP-1	This study
BAX-9/pHY-Pfen/BSP-1	BAX-9 harboring the plasmid pHY-Pfen/BSP-1	This study
BAX-9/pHY-Pbac/BSP-1	BAX-9 harboring the plasmid pHY-Pbac/BSP-1	This study
*B. subtilis* 168	the strain containing P43 promoter	Stored inlab
*B.licheniformis* WX-02	CCTCC M208065, wild type	Stored inlab
**Plasmid**	**Characteristics**	**Source**
pHY300PLK	*E. coli−Bacillusshuttle* vector for geneexpression, Apr, Tet	Stored in lab
pHY-PX/BSP-1	pHY300PLK + *bsp-1*+ TamyL	This study
pHY-Psra/BSP-1	pHY300PLK + Psra + *bsp-1*+ TamyL	This study
pHY-Psrs/BSP-1	pHY300PLK + Psrs + *bsp-1*+ TamyL	This study
pHY-PrnpB/BSP-1	pHY300PLK + PrnpB + *bsp-1*+ TamyL	This study
pHY-Pffs/BSP-1	pHY300PLK + Pffs + *bsp-1*+ TamyL	This study
pHY-Phyp/BSP-1	pHY300PLK + Phyp + *bsp-1*+ TamyL	This study
pHY-Ppqq/BSP-1	pHY300PLK + Ppqq + *bsp-1*+ TamyL	This study
pHY-Pscp/BSP-1	pHY300PLK + Pscp + *bsp-1*+ TamyL	This study
pHY-Pcsp/BSP-1	pHY300PLK + Pcsp + *bsp-1*+ TamyL	This study
pHY-P43/BSP-1	pHY300PLK + P43+ *bsp-1*+ TamyL	This study
pHY-PtrnQ/BSP-1	pHY300PLK + PtrnQ + *bsp-1*+ TamyL	This study
pHY-Psrf/BSP-1	pHY300PLK + Psrf + *bsp-1*+ TamyL	This study
pHY-Pitu/BSP-1	pHY300PLK + Pitu + *bsp-1*+ TamyL	This study
pHY-Pfen/BSP-1	pHY300PLK + Pfen + *bsp-1*+ TamyL	This study
pHY-Pbac/BSP-1	pHY300PLK + Pbac + *bsp-1*+ TamyL	This study

**TABLE 2:** Primers used in this study.

Primer name	Sequence of primer (5' to 3')
*bsp-1*-F	CG**GGA​TCC**ATG​AGA​AGC​AAA​AAA​TTG​TGG​AT
*bsp-1*-R	TTA​TTG​TGC​AGC​TGC​TTG​TAC​G
*bsp-1* (Ppqq)-F	ACA​GCT​TCA​TTG​CGA​ATG​AGA​AGC​AAA​AAA​TTG​TGG​AT
*bsp-1* (Tamyl)-R	AAG​AGC​AGA​GAG​GAC​TTA​TTG​TGC​AGC​TGC​TTG​TAC​G
TamyL-F	GCA​GCT​GCA​CAA​TAA​AAG​AGC​AGA​GAG​GAC​GGA​TT
TamyL-R	GC**TCT​AGA**CGC​AAT​AAT​GCC​GTC​GCA​CT
Psra-F	CG**GAA​TTC**AAC​GAA​AAG​ACG​CCA​AAA​G
Psra-R	CG**GGA​TCC**TTG​TTA​AGG​GTA​TAC​GGG​AGA​T
Psrs-F	CG**GAA​TTC**GCA​GTT​TGT​TTC​TTG​AAA​ATC​A
Psrs-R	CG**GGA​TCC**CAC​AGC​AGA​TTT​TGA​TTT​TCA​A
PrnpB-F	CG**GAA​TTC**GGT​CGT​ATT​CGG​CGC​AT
PrnpB-R	CG**GGA​TCC**CAA​AAT​AAA​TAT​CAA​ATT​TTG​ATA​TG
Pffs-F	CG**GAA​TTC**GGA​TTA​TGA​AAC​CTT​TCA​TCA​AG
Pffs-R	CG**GGA​TCC**TAA​GAA​CAC​TTG​TTC​ATT​ATA​AAG​C
Phyp-F	CG**GAA​TT**CTT​AAA​ATC​ACA​CTG​ACA​GCA​GAC
Phyp-R	CG**GGA​TCC**CTG​CCA​TTT​GTT​CTC​ACC​TC
Ppqq-F	CG**GGA​TCC**GGC​AGG​AGC​TGT​CTC​TTT​AT
Ppqq-R	TTT​TTT​GCT​TCT​CAT​TCG​CAA​TGA​AGC​TGT​CTT​T
P43-F	CG**GAA​TTC**TGA​TAG​GTG​GTA​TGT​TTT​CG
P43-R	CG**GGA​TCC**GTG​TAC​ATT​CCT​CTC​TTA​CCT​ATA​ATG
PtrnQ-F	CG**GAA​TTC**GTC​GTC​TCT​TTT​TCC​CAT​TTT
PtrnQ-R	CG**GGA​TCC**ATA​TAG​ACT​GCG​TTA​TGA​GAA​CGT​C
Pscp-F	CG**GAA​TTC**CTA​AAA​AAT​AGT​GAT​TTT​TAT​CAG​G
Pscp-R	CG**GGA​TCC**ATC​TAT​TCC​TCC​TTT​TCT​TTT​ACT​A
Pcsp-F	CG**GAA​TTC**AAC​ATG​TTA​TTT​CGA​AAA​AAG​TTA
Pcsp-R	CG**GGA​TCC**GAA​ATT​TCC​TCC​TAA​AGC​GAC
Psrf-F	CG**GAA​TTC**AGC​GCT​CTA​TGT​AAA​ATA​GAG​TGC
Psrf-R	CG**GGA​TCC**ATG​TGT​GCG​CCT​CCC​CTT
Pitu-F	CG**GAA​TTC**TAA​TTT​CTG​ACA​CAA​TAA​TGC​CAA
Pitu-R	CG**GGA​TCC**GAG​ATT​CCT​CCG​ATC​ATA​TTG​AA
Pfen-F	CG**GAA​TTC**CAA​AAA​TGG​GCG​GAA​TTT​T
Pfen-R	CG**GGA​TCC**AAT​GGC​AGT​TTT​ATC​CTC​CAG
Pbac-F	CG**GAA​TTC**ATT​CAT​TCA​CAT​CCT​CCT​TAA​GA
Pbac-R	CG**GGA​TCC**CTG​GAT​TTC​CCC​GCC​TT
pHY300-YF	GTT​TAT​TAT​CCA​TAC​CCT​TAC
pHY300-YR	CAG​ATT​TCG​TGA​TGC​TTG​TC

Note: Restriction sites highlight in bold.

### 2.3 Skim milk agar medium assay

Skim milk agar medium contain 10 g/L tryptone, 5 g/L yeast extract, 10 g/L NaCl, 1.5% Agar and 2% skim milk. To screen the protease activity, 20 µL of supernatant of the cultures were added to the wells of milk agar plates made by a hole punch. The radius of transparent circles were measured after the plates were cultured at 37°C for 12 h.

### 2.4 Protease fermentation

The *B. amyloliquefaciens* cells were inoculated into 5 ml liquid LB medium (10 g/L tryptone, 5 g/L yeast extract, and 10 g/L NaCl), and incubated at 37°C for 12 h with shaking at 180 rpm. Then, a 3% (v/v) inoculum was transferred into the 50 ml alkaline protease fermentation medium (40 g/L tryptone, 20 g/L yeast extract, and 10 g/L NaCl) and cultured at 37°C for 72 h with shaking at 180 rpm.

### 2.5 SDS-PAGE analysis of the expressed proteins

Protein samples were prepared by 100% trichloroacetic acid (TCA) precipitation (Benabdelkamel et al., 2018). Specifically, 0.90 ml of fermentation supernatant was mixed with 0.10 ml of trichloroacetic acid, inverted 10 times to mix and incubated at 4°C for 12 h. Then, the mixture was centrifuged at 6,000 ×*g* for 10 min. After that, the supernatant was discarded and the pellet was washed with 0.20 ml of absolute ethanol for three times. After dried at 37°C, the pellet was dissolved with 30 µL of loading buffer containing 8 mol/L urea and 2 mol/L thiourea. At last, the samples were analyzed by SDS-PAGE electrophoresis.

### 2.6 Enzyme activity analysis

1 ml of the sample was transfered to tube A (as blank control) and tube B for activity measurement, respectively. After the samples were heated at 40°C for 2 min, A was supplemented with 2 ml of trichloroacetic acid (64.70 g/L), while B was added with 1 ml of casein solution (10 g/L). Both samples were further heated at 40°C for 10 min. Then, 1 ml casein solution was added into A, and 2 ml trichloroacetic acid was added into B. The mixtures were placed at room temperature for 20 min. Subsequently, A and B were filtered and obtained supernatant, and added 5 ml of sodium carbonate solution and 1 ml of folin phenol reagent to the supernatant, mix well and heat at 40°C for 20 min, and measure the absorbance at 680 nm wavelength. The activity 1) of the dilution of sample was obtained from the standard curve. The enzymatic activity of the sample was calculated as follows:

$$\mathrm{Enzyme}\;\mathrm{activity}\left(U/\mathrm{mL}\right)=\frac{a\times 4\times N}{10}\;\left(N:\mathrm{Dilution}\;\mathrm{factor}\;\mathrm{of}\;\mathrm{sample}\right)$$

### 2.7 Transcriptional analysis during the alkaline protease fermentation

The recombinant engineered strains were cultured for 12 h, and then the cells were collected by centrifugation and washed for transcription analysis, according to the description of Total RNA Isolation Kit and PrimeScript RT Master Mix Kit. DNA removal and RNA reverse transcription were performed simultaneously in one system. The reaction conditions which included EasyScript^®^ RT/RI and gDNA Remover were 25°C for 10 min, 42°C for 15 min, and 85°C for 10 s. RT-PCR amplification was performed using three-steps method. Pre-denaturation: 1 cycle (95°C for 5 min); PCR reaction: 40 cycles (95°C for 30 s, 60°C for 30 s) and dissolution curve (1 min at 95°C, 1 min at 65°C). Relative quantitative analysis was performed with 16 s RNA as the internal reference gene.

### 2.8 Statistical analysis

Each group of experiments was designed with three independent replicates. SPSS 20.0 was used for statistical analysis, calculating the means and standard deviations, and evaluating the significance. Origin 8.5 was used to process the data and make the graphs.

## 3 Results and discussion

### 3.1 Identification of an alkaline protease from a new *B. subtilis* strain

**FIGURE 1:**
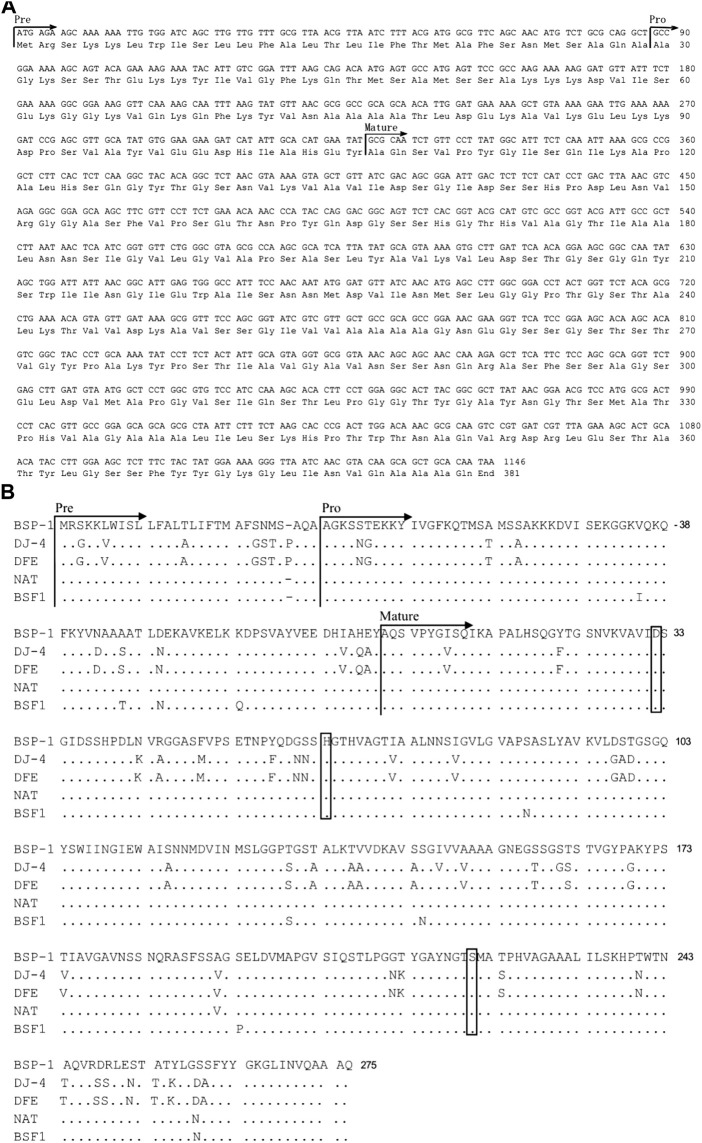
Sequence analysis of *bsp-1* gene and deduced amino acids. **(A)** Nucleotide (upper line) and deduced amino acid (lower line) sequences of the protease BSP-1. The predicted signal peptide, propeptide and mature peptide are marked with arrows. **(B)** Amino acid sequence alignment of the protease BSP-1, with subtilisin (DJ-4, DFE) from *B. amyloliquefaciens* and subtilisin (NAT, BSF1) from *B. subtilis*. The signal peptide, pro-peptide, and mature peptide (mature) were indicated with arrows. “.” indicated the same residue as the first sequence. The catalytic center residues (Asp-32, His-64, and Ser-221) were boxed. The initial amino acid of the mature peptide was numbered as + 1.

We isolated a *B. subtilis* strain with high protease activity using skim milk plates. Then the new *B. subtilis* strain was named *B. subtilis* D7. In order to identify an alkaline protease from *B. subtilis* D7, we designed a primer set using the gene sequence of subtilisin NAT (S51909) from *B. subtilis*. With the primers, we amplified a gene fragment from *B. subtilis* D7. Sequencing of the gene fragment indicates that it encodes an alkaline protease (BSP-1) containing 381 amino acids (aa) (Figure 1A). Analysis of the protein sequence using SignalP 5.0 indicates a 29-aa predicted signal peptide, while the 30-77 aa and 78-275 aa are the propeptide and mature peptide, respectively (Figure 1A). We aligned the sequence of BSP-1 with four well-characterized proteases (Choi et al., 2004; Yong et al., 2004; Agrebi et al., 2009; Ku et al., 2009), indicating that it has the highest similarity to subtilisin NAT (S51909) and fibrinolytic enzyme BSF1(FJ517584) (>96% similarity) and less similar to subtilisin DJ-4 and subtilisin DFE (Figure 1B). In addition, all the proteases contain the conserved catalytic triad, including Asp-32, His-64, and Ser-221. Together, the data indicates that BSP-1 belong to the subtilisin family of serine proteases (Wei et al., 2011).

### 3.2 Heterologous expression of BSP-1 in *B. amyloliquefaciens* BAX-9

**FIGURE 2:**
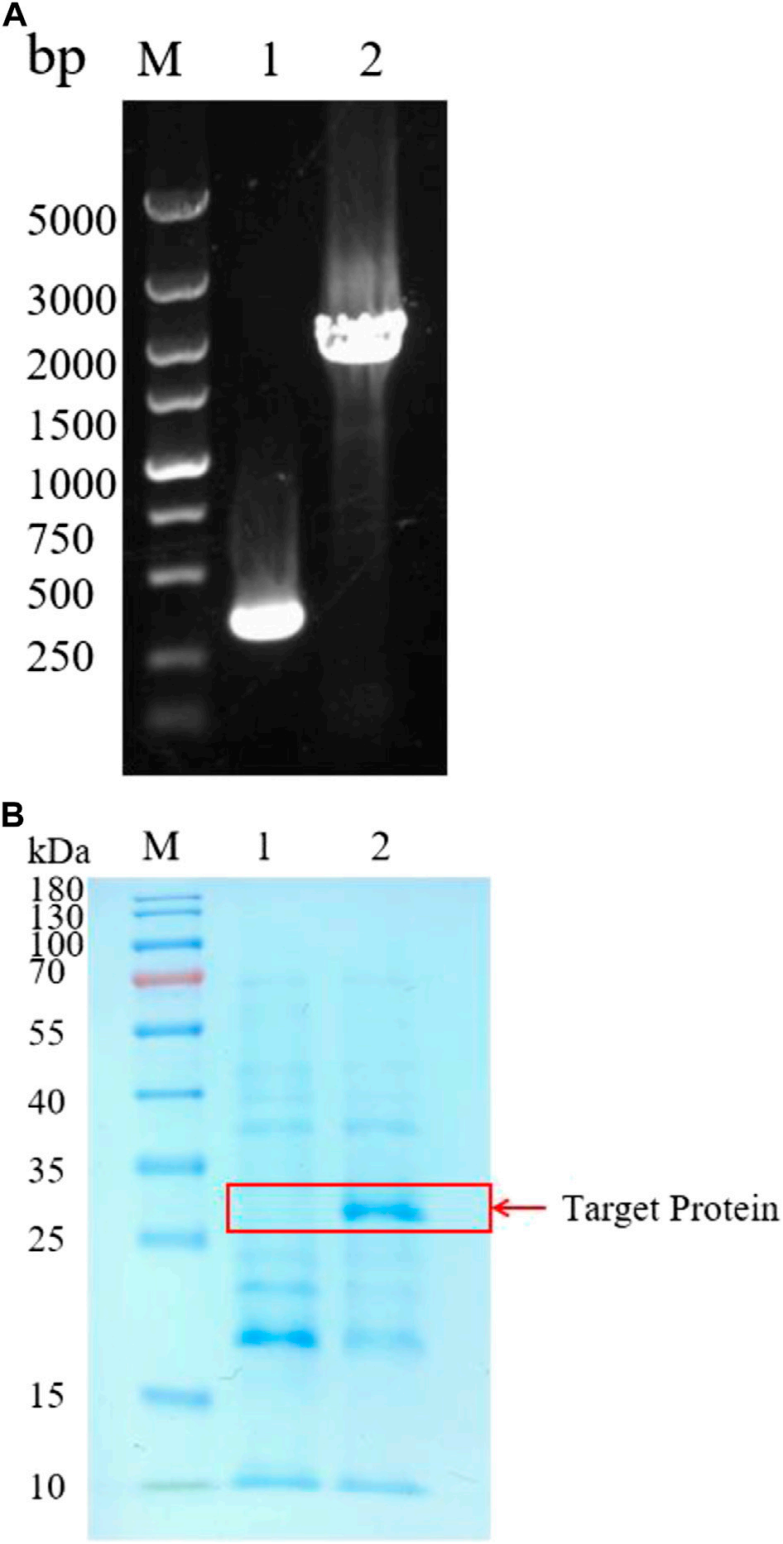
Heterologous expression of protease gene *bsp-1* in *B amyloliquefaciens* BAX-9. **(A)** PCR analysis of recombinant strain colonies. M: Marker; 1-2: P BAX-9/pHY-300 and BAX-9/pHY-Ppqq/BSP-1. **(B)** SDS-PAGE of analysis of BAX-9/pHY-Ppqq/BSP-1. M: Marker; 1-2: BAX-9/pHY-300 and BAX-9/pHY-Ppqq/BSP-1.

*B. amyloliquefaciens* is considered as a safe strain that have been widely used to express industrial enzymes. *B. amyloliquefaciens* is rich in proteases, and can degrade heterologous proteins. Knockout of the protease genes may solve the problem. Therefore, we expressed BSP-1 in *B. amyloliquefaciens* BAX-9, a strain where many protease genes (*epr, nprE, aprE, aprX, mpr, bpf, vpr, htrB, yktc1*) have been knocked out (Chen et al., 2022). At first, the promoter of PQQ-binding-like beta-propeller repeat gene (Ppqq) from *B. amyloliquefaciens* was used to express BSP-1. The constructed expression plasmid, pHY-Ppqq/BSP-1, was electroporated into *B. amyloliquefaciens* BAX-9, generating the strain BAX-9/pHY-Ppqq/BSP-1 (Figure 2A). Subsequently, the fermentation broth of BAX-9/pHY-Ppqq/BSP-1 and the control strain (BAX-9/pHY-300) were used for SDS-PAGE analysis. As shown in Figure 2B, compared to the control strain (BAX-9/pHY-300), the target protein (30 kDa) was found in the fermentation broth of BAX-9/pHY-Ppqq/BSP-1. This result demonstrates that the *bsp-1* gene was significantly expressed in BAX-9. The thickness of the band corresponds to the amount of protein present, the thicker band of the target protein also shows the feasibility and great potential of expressing the heterologous protein in *B. amyloliquefaciens*.

### 3.3 Promoter screening enhanced BSP-1 expression

To further improve the expression of BSP-1 in *B. amyloliquefaciens,* we screened different promoters driving the expression of BSP-1. The promoters include two reported high-strength promoters (P43 (Wang and Doi, 1984), PtrnQ (Song et al., 2016)) from *B. subtilis* and a promoter collection from *B. amyloliquefaciens* HZ-12. We selected the promoters of potentially highly-expressed genes in HZ-12, including *ssrA* (NC_014551.1: 3271400-3271038)*, ssrS* (NC_014551.1: 2609656-2609464)*, rnpB* (NC_014551.1: 2202786-2202405)*, ffs* (NC_014551.1: 27641-27905)*,* cold-shock protein gene (NC_014551.1: 1029767-1029567), PQQ protein gene (NC_014551.1: 3859752-3858532), spore coat protein gene (NC_014551.1: 1272412-1271912), hypothetical protein gene (NC_014551.1: 1272819-1272505). In addition, since *B. amyloliquefaciens* is highly productive of macromolecular substances such as lipopeptides, the promoters of four gene clusters (Surfactin, Iturin A, Fengycins, Bacillaene) producing lipopeptides, i.e., Psrf, Pitu, Pfen, Pbac, respectively, were also used in the study.

**FIGURE 3:**
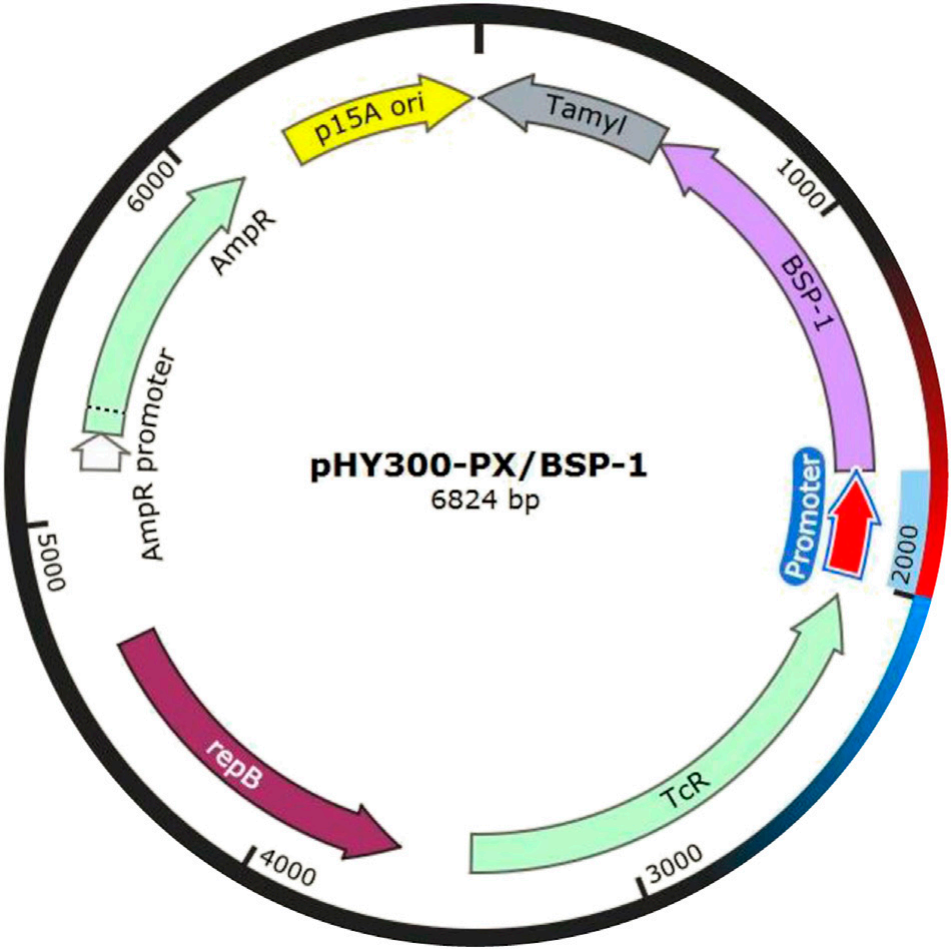
Illustration of pHY300-PX/BSP-1, where the promoter sequence can be easily replaced.

**FIGURE 4:**
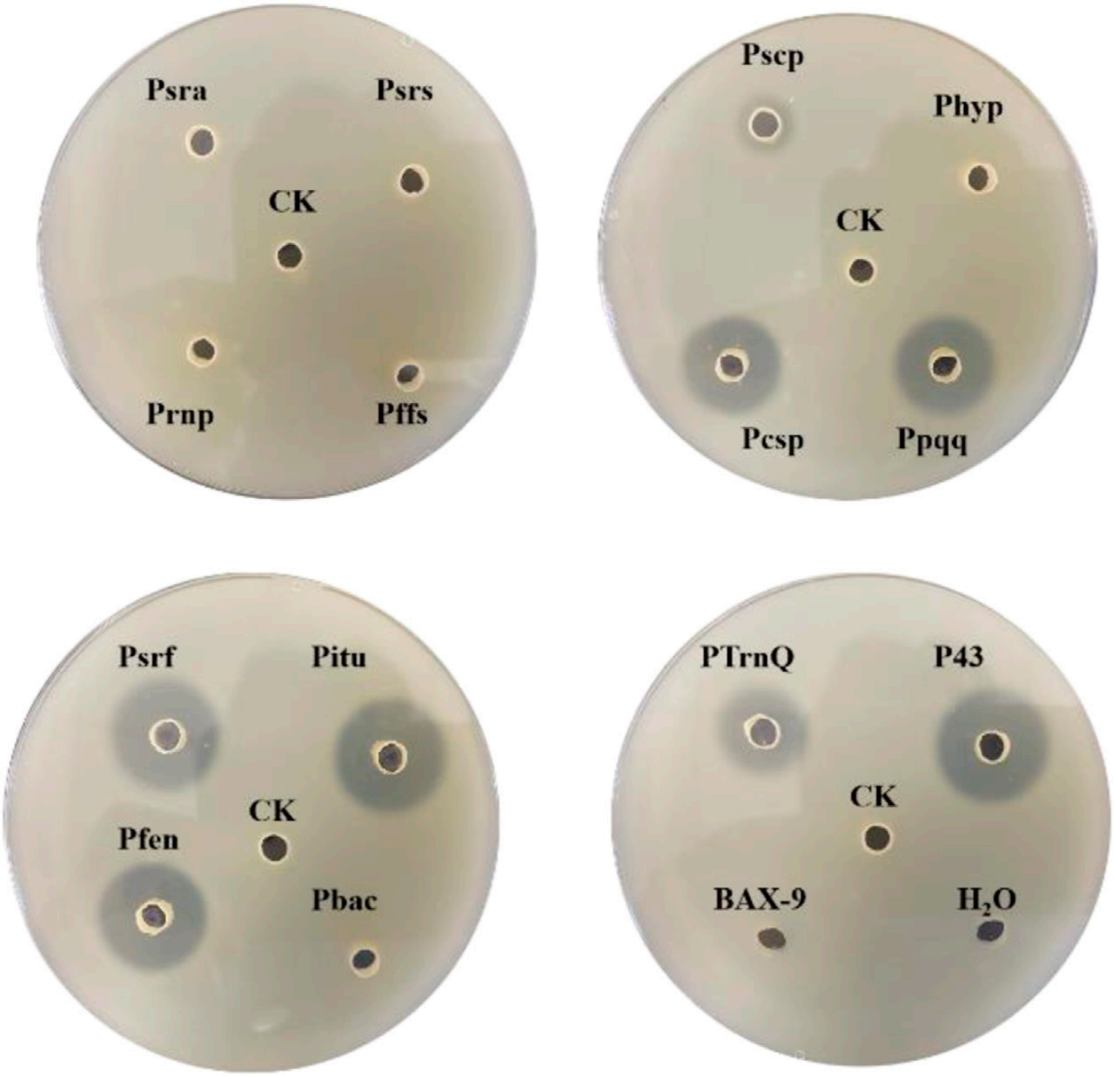
Skim milk agar plates of the fermentation supernatant of different strains. CK: BAX-9/pHY-300, Psra: BAX-9/pHY-Psra/BSP-1, Psrs: BAX-9/pHY-Psrs/BSP-1, Prnp: BAX-9/pHY-Prnp/BSP-1, Pffs: BAX-9/pHY-Pffs/BSP-1, Pcsp: BAX-9/pHY-Pcsp/BSP-1, Ppqq: BAX-9/pHY-Pscp/BSP-1, Pscp: BAX-9/pHY-Pscp/BSP-1, Phyp: BAX-9/pHY-Phyp/BSP-1, Psrf: BAX-9/pHY-Psrf/BSP-1, Pitu: BAX-9/pHY-Pitu/BSP-1, Pfen: BAX-9/pHY-Pfen/BSP-1, Pbac: BAX-9/pHY-Pbac/BSP-1, P43: BAX-9/pHY-P43/BSP-1, PtrnQ: BAX-9/pHY-PtrnQ/BSP-1.

For efficient insertion of the promoters, we constructed a plasmid with replaceable promoter sequence by inserting the fusion fragment of *bsp-1* gene and TamyL terminator into pHY300PLK, resulting in the plasmid pHY300PLK-PX/BSP-1 (Figure 3). Then, the sequences of the promoters were amplified and then inserted into pHY300PLK-PX/BSP-1. The resulting plasmids were transformed into BAX-9 to generate engineered strains for further analysis. Engineered strains containing different promoters (Psra, Psrs, Prnp, Pffs, Ppqq, Pcsp, Pscp, Phyp, Psrf, Pitu, Pfen, Pbac, P43, PtrnQ) and the control strain (BAX-9/pHY-300, BAX-9) were fermented. And the enzyme activities of these strains were preliminarily analyzed by using skim milk agar plates. As shown in Figure 4, the supernatant of the fermentation broth of the engineering strains containing P43, Pitu, Pfen, Ppqq, Psrf, Pcsp, PtrnQ and Pscp produced transparent circles of different radius (7, 7, 7, 6.50, 6, 6, 4, 1.50 mm, respectively), while other promoters (Psra, Psrs, Prnp, Pffs, Phyp, Pbac) did not produce transparent circles. The size of the transparent circle reflects the activity of BSF-1, the size of the transparent circle is positively correlated with the activity.

**FIGURE 5:**
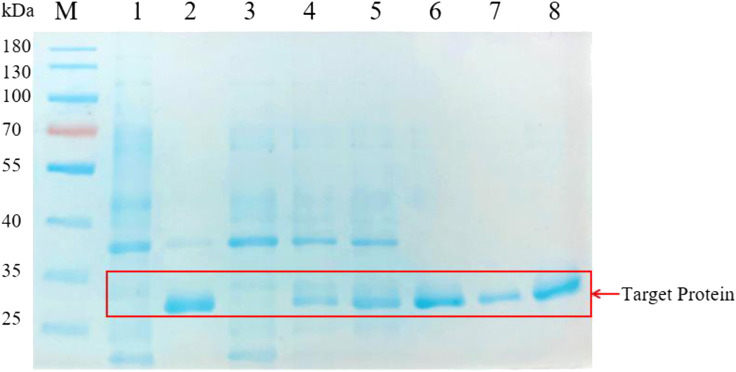
SDS-PAGE analysis of the fermentation supernatant of different strains. M: Marker; 1-8: BAX-9/pHY-300, BAX-9/pHY-P43/BSP-1, BAX-9/pHY-Pscp/BSP-1, BAX-9/pHY-Pcsp/BSP-1, BAX-9/pHY-Ppqq/BSP-1, BAX-9/pHY-Psrf/BSP-1, BAX-9/pHY-Pitu/BSP-1, BAX-9/pHY-Pfen/BSP-1.

Accorded to the result of the transparent circle, we selected the Pscp, Pcsp, Ppqq, Psrf, Pitu, Pfen promoters from *B. amyloliquefaciens* and the P43 promoter from *B. subtilis* for further analysis. We analyzed the proteins in the supernatant of BAX-9/pHY-Pscp/BSP-1, BAX-9/pHY-Pcsp/BSP-1, BAX-9/pHY-Ppqq/BSP-1, BAX-9/pHY-Psrf/BSP-1, BAX-9/pHY-Pitu/BSP-1, BAX-9/pHY-Pfen/BSP-1 and BAX-9/pHY-P43/BSP-1 by SDS-PAGE. As shown in Figure 5, BSP-1 was successfully expressed in all recombinant strains, and exhibited the highest level under the promoter P43, Psrf and Pfen, which was consistent with the result of the transparent circle. The data indicates that a high-strength promoter facilitates the protein expression level and thus increase the activity outcome of protein.

### 3.4 Effects of different promoters on the activity of BSP-1

**FIGURE 6:**
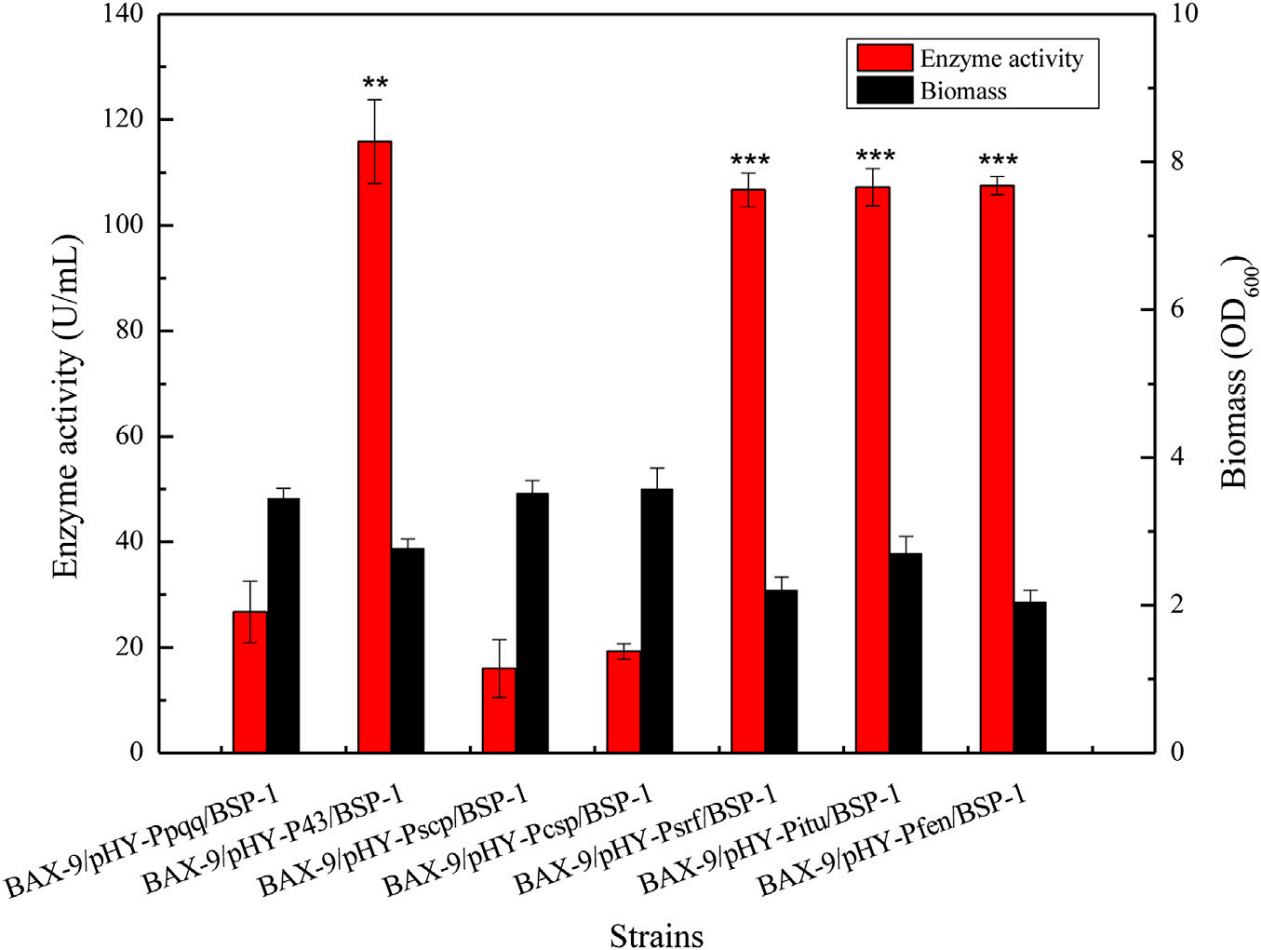
Determination of enzyme activity and biomass of recombinant strains. Asterisks show the significant difference (*p* < 0.05) compared with the control.

To further analyze the effect of different promoters on the expression level of protease gene *bsp-1*, we measured the activity of BSP-1 and the biomass of the seven cultures at 48 h. As shown in Figure 6, the activity of BSP-1 expressed by the promoter of P43, Psrf, Pitu, and Pfen reached 115.87, 106.71, 107.25, and 107.52 U/mL respectively, improving by 4.3-fold, 4.0-fold, 4.0-fold and 4.0-fold compared to the Ppqq promoter (26.71 U/mL). The data indicated protomer sceening efficiently enhanced the activity outcome of BSP-1. In addition, the biomass of Ppqq promoter was significantly higher than that of P43, Psrf, Pitu and Pfen promoter, indicating that the enzyme activity and the biomass are not necessarily positively correlated.

### 3.5 Verification of the transcription of *bsp-1* drived by different promoters

**FIGURE 7:**
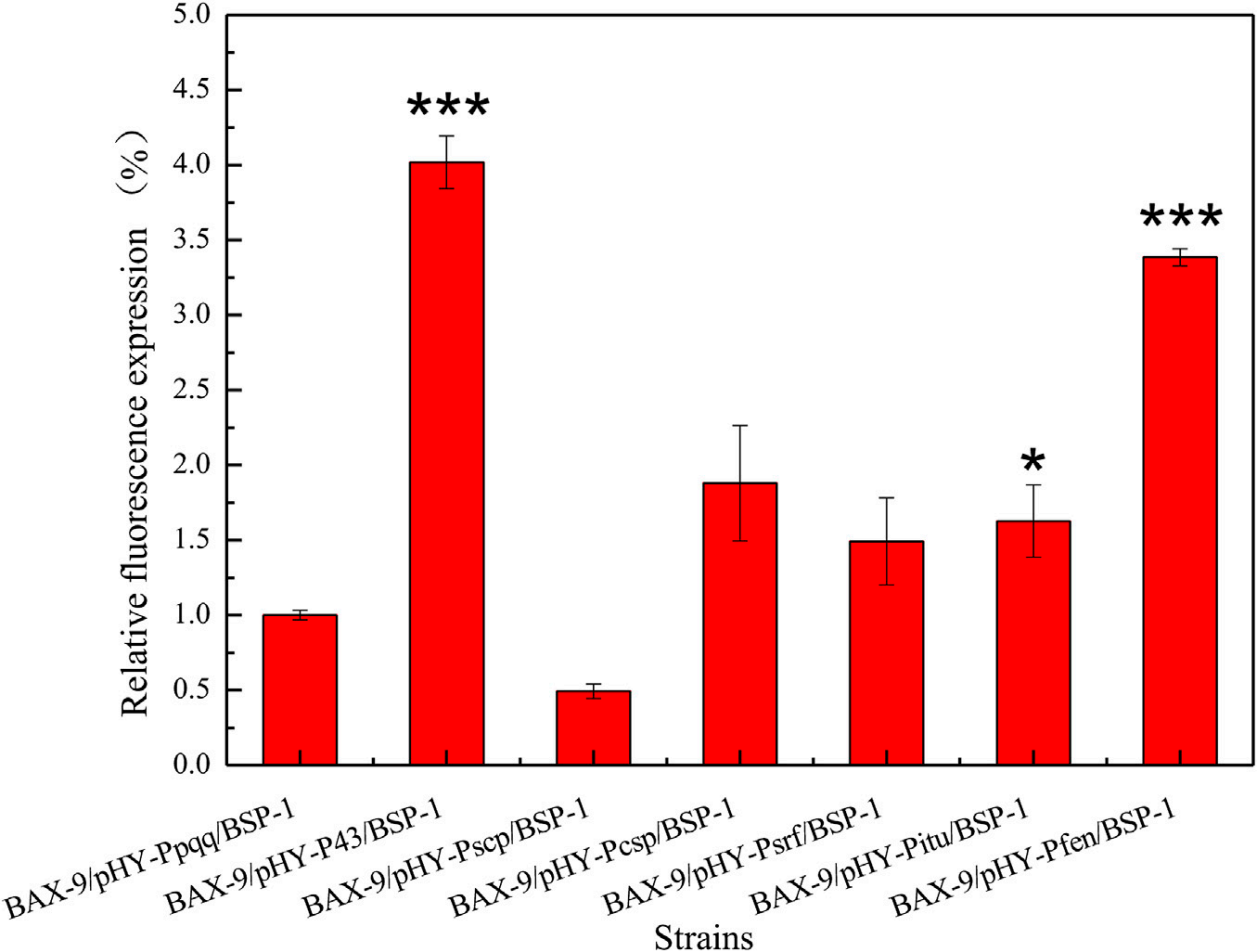
RT-PCR results of recombinant strains. Asterisks show the significant difference (*p* < 0.05) compared with the control.

To verify the effect of promoter on gene transcription level, we established a microbial fermentation model. The total RNA of different recombinant strains was extracted and analyzed. As shown in Figure 7, the transcription levels of *bsp-1* in BAX-9/pHY-P43/BSP-1, BAX-9/pHY-Pscp/BSP-1, BAX-9/pHY-Pcsp/BSP-1, BAX-9/pHY-Psrf/BSP-1, BAX-9/pHY-Pitu/BSP-1, BAX-9/pHY-Pfen/BSP-1 significantly increased compared to that in BAX-9/pHY-Ppqq/BSP-1. Among them, BAX-9/pHY-P43/BSP-1 showed the highest transcription level, 4 folds than that in BAX-9/pHY-Ppqq*/*BSP-1. The results of RT-PCR were consistent with the results of SDS-PAGE and enzyme activity, revealing that the efficient transcription by P43 generated high protein level and enzyme activity. The above results provide a reference to select the optimal promoter for protein expression.

### 3.6 The optimization of fermentation for BSP-1 production in BAX-9/pHY-P43/BSP-1

**FIGURE 8:**
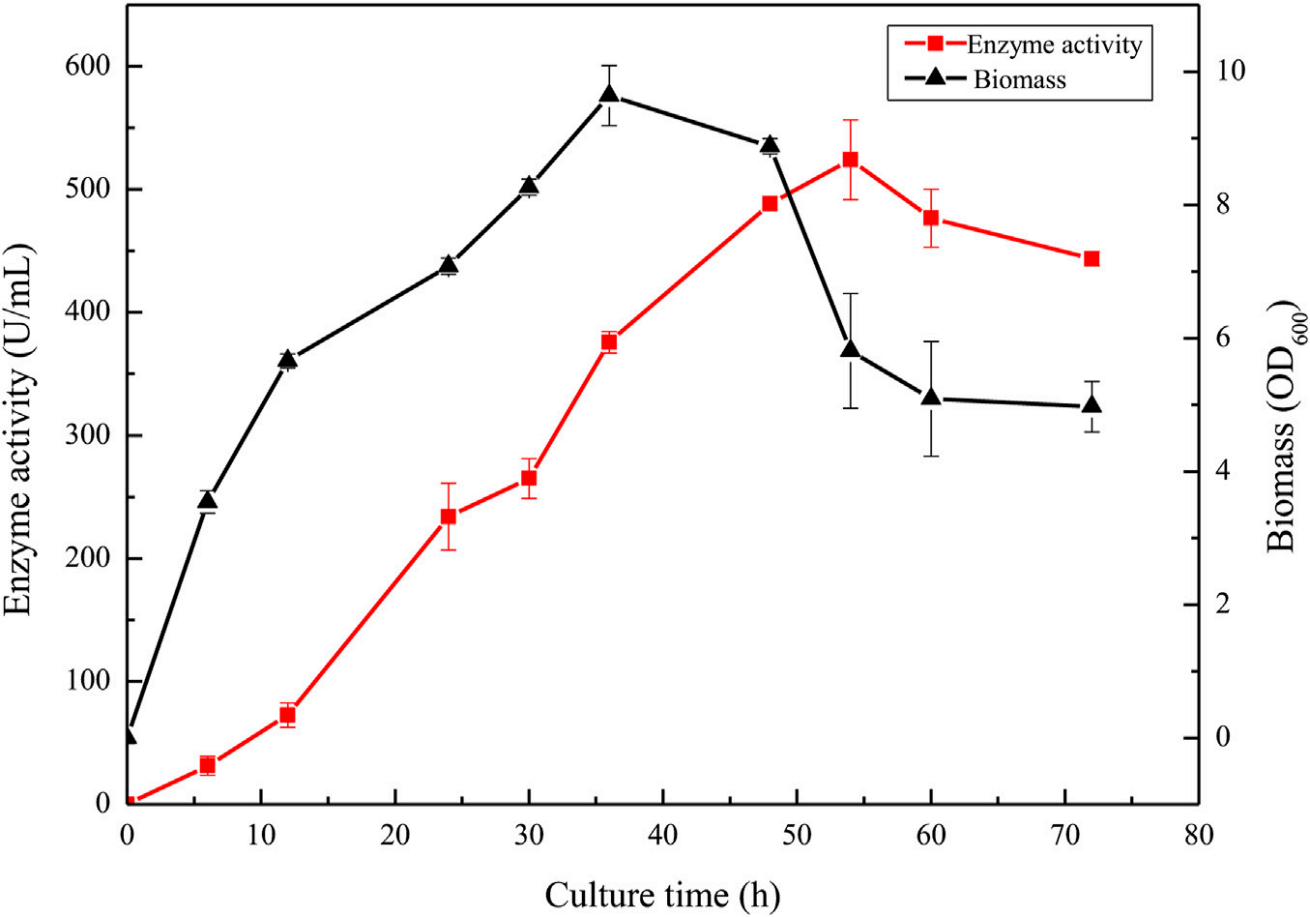
Enzyme activity and culture growth of BAX-9/pHY-P43/BSP-1 under fermentation using the optimized fermentation medium.

To further improve the protease yield of the recombinant strain BAX-9/pHY-P43/BSP-1, an optimized medium (40 g/L tryptone, 20 g/L yeast extract, and 10 g/L NaCl) was selected for fermentation (Supplementary Figure S1). During the fermentation process, the activity of BSP-1 protease and the biomass of strains were determined. As shown in Figure 8, in the initial 36 h, the enzyme activity increased as the culture was growing. With the continuation of fermentation, the biomass began to decrease, and the enzyme activity continued to increase until 54 h. The enzyme activity reached a maximum of 524.12 U/mL. Finally, with the continuous decrease of biomass, the enzyme activity declined and tended to be stable. The results indicate that the accumulation of the enzyme was delayed compared to culture growth.

## 4 Conclusion

In conclusion, the BSP-1 protease gene from *B. subtilis* was identified in this study, and explored possibility of high-efficiency expression system via *B. amyloliquefaciens*. The enzyme activity and transcription level under the expression of different promoters Ppqq, Pscp, Pcsp, Psrf, Pitu, Pfen and P43 were analyzed, and we found P43 was the optimal promoter for heterologous expression of BSP-1 protease. Nevertheless, Psrf and Pfen show comparable expression strength to P43 and thus can be used as high-strength promoters in future. After fermentation optimization, the highest enzyme activity of the engineering strain BAX-9/pHY-P43/BSP-1 reached 524.12 U/mL. Moreover, monitoring the fermentation process also confirmed no direct correlation between enzyme activity and biomass. This study enriched the originals and hosts for protein expression, and it identified a new resource for the industrialized production of proteases.

## Data Availability

The original contributions presented in the study are included in the article/Supplementary Material, further inquiries can be directed to the corresponding author.
