# Supplementary material for: Genetic engineering for enhanced production of a novel alkaline protease BSP-1 in Bacillus amyloliquefaciens
Source: Front Bioeng Biotechnol. 2022 Aug 30;10:977215. doi: 10.3389/fbioe.2022.977215 (PMC9468883; doi:10.3389/fbioe.2022.977215)
Supplement: Supplementary file 1 [file DataSheet1.PDF]

Supplementary Material

Nucleic acids sequences of promoters involved in this study.

Psra:
AACGAAAAGACGCCAAAAGAGAAATCGAAAGAGCGTTCAGAGACAGACAAAAAGGCTTCTGATGCCTGA
GCCTTGATGTGATGATATGCCCTGTGCTATACTGTTCACACATCAGATTCACAGGCTGTTTATTGAAGATAT
TCCCAATTGAATTTGCCTGTGATCATGGTATAATAACAGTACAGCCAGTTGAGCAAGAGCTTGATAGATCT
CCCGTATACCCTTAACAA

Psrs:
GAGATACGATCGCTTTCCCGAAAACGGCAAGCGCAAGCTGCCTTCTTACGGAAGCGCCCGGCGAAGTGAG
CGACGCCCAGCTTGAAGAACTTCACCTTTCTGTCAAAAGAAAAGTGAAACAGTAAAGATCGGCAGTTTGTT
TCTTGAAAATCAAAATCTGCTGTGATATGATTTTGACAAGTTAAAT

PrnpB:
GGTCGTATTCGGCGCATTTGATCCCAAGGGGGGCTGTACAGGAACGCTGATGAACCTCTTGCAGGAAGAGC
GGTTCAACCACCAAGCGGAAGTGGTGAGCGGCGTGCTCGGAGAAGAGTGCGGAGAAATGCTCAGCGCTTT
TTTCAGAAAGCTGCGCCGGCAAAAGAAAGACATGAGGAAAAAGTTGTCTGAGTAGTACGGTTGCATTTTCT
GCCTGAAGCAGATATACTTATGTGTGCATCATGAATGGTGCGCATATCAAAATTTGATATTTATTTTG

Pffs:
GGATTATGAAACCTTTCATCAAGAAATTGAAGAACTGCAGCAGGAAAATCTGCAGCTGAAAAAACAGCTT
GAAGAAGCAAGCAAAAAACAGCCGGTGCAGTCCAATACGACCAACTTTGATATTCTGAAAAGGCTGTCCA
ACCTTGAGAAACATGTATTCGGCAGCAAACTTTATGACGAATAAGGCAGATGCACTTGTCAAATGAGACAA
GTTGCTTTATAATGAACAAGTGTTCTTA

Pscp:
CTAAAAAATAGTGATTTTTATCAGGATAGATGCCTGAGTCAAAATAAGGGGCCGGCTCATTTAATAATAGT
AAAAGAAAAGGAGGAATAGAT

Phyp:
TTAAAATCACACTGACAGCAGACGATATCATGGCCTTTGTACAAATTTTGTTTCAATTATTAAATATACTTT
TTGCTGAACTAGACATCCTTTAACCTTTCTTTTCAAATATTTAAATAGAGGTGAGAACAAATGGCAG

Pcsp:
AACATGTTATTTCGAAAAAAGTTACATTTAGCAGTTGTTTTTTCTTCGGATTACTGGTAGAGTAAAGGTAAT
TATTTTTGTTCGAACTATCTTTAAGAAGAAAGTTTTGTAAGAGTTTTCGTCTTGGAAGTTTGTTTTAGAGCAA
GAATAGTGAATTTAAGCGTTTGTCGCTTTAGGAGGAAATTTC

Ppqq:
GGCAGGAGCTGTCTCTTTATTACGGTCTTTTCGTTTGGTCAGGCGCGGCTTTCATCGTCAATGTGGTGTGGT
ATATTGCCGCGTTTCGATCATCCCCGAAGCATCAGCAGGCTATGTGACATTTGTTCTATAAATCTACCGAAA
ATACAAATATTTCATAGGATGATTATGGTATGATGAAAGTGGTATAGCAGTTTTTAATAGAAACGGGAGGA
ACTGACATTGAAAAAAAAGACAGCTTCATTGCGA

Psrf:
AGCGCTCTATGTAAAATAGAGTGCTTTTTTTGCGGTTTAATGAAATCATATTGCGAACGATTAAATCTCCTG
TTTCGGTTCTTGCGCTGTTTTTTCCCCATCAGCTCATATATGAAAACTATTTTCATTCTGCCATAACTGGATA
TTCGAGAGATTTATACTATAGTTTAAAGATTTTTAATTTTACATAAATAATATTTTTAAAAATAAATTGCGG
GATGCCGCAAAATACCCTCTGGAATTGTCGGAATTTTTTTCGGTGTGCCGAATGAAACTTTTCACCCATTTT
TCGGTGATAAAAACAATAATTTCATATAAAGTGAACGTAAGTAGATATATAGTAATTCATGAAGAAATAGG
TAAACCCTGTTGCTTACAAACATTGGATTCTTGTCTTGATTTCTCATAAATTTGAGCCGCATTTCGGACTGTG
CGGGTGAGTGGATTGCGGATTTCGGCGGTGATTGAATCGGGATCGTTTTGGAGGTAAGTGGTTCTTTTGGCT
TGATAATGAGTTAGGGACATTGAGGGAGGCTGTTTCTAAGGGAGAATTGACAATTTTATCTTAAAAAGGGG
AGGCGCACACAT

Pitu:
TAATTTCTGACACAATAATGCCAATAGCCCTAAAACATATGAAACATGAAGAGCGCGACCTAAGTGATGAA

GAGCCGGTAACAGGCTCCACTCCGGCTTTTCATCATCTCTCTTTAAGACTGATTTTCGGTGAAACCCCATGT
TTTATTTTATGAATAAATATATCTAATTCGTTTGTAAAATCGAACAATAACTCCTCCGAAAGTATTCTATAC
ACATTTGTTATAATCATGCTAGGATGTTAGATAAAGGGATAATTTGTAGGATATTGTATTTCTGTTCAATAT
GATCGGAGGAATCTC

Pfen:
CAAAAATGGGCGGAATTTTTCACGACACTTGCCGAAAACGCCGCAGCTGATCCGGGTTTGGAATTAGACGA
TATTTCTGTTTTGTCAGAAAAAGAAGAAGTGTCTCTGCTTCAAAATTTCAGCCCCTTGCAGAAAACAGCGTT
TCCGCTTCATCAGCCTCTTCATGAGCTGCTGGAACAACAGGCGGAAAAAACGCCGGACCGTCCGGCGATTT
TAACTGATGACATTTCGATTACGTATCAGGAGCTGAACGAAAGAGCGAATGAGCTTGCGCACCGGCTGATC
AAGCGGGGAATAAGGCTGGAGGATAAAACTGCCATT

Pbac:
ATTCATTCACATCCTCCTTAAGAAGTTGATATGAAGCGTAAATATGTATATTAGCCGGATTTAACACACATG
ATGAAATGGCAAAAAAAGTTTGCTGTTTTGTTGATAATCAACGAAAATGGCGGCTTTACGATTTGTCTATTG
AACCGGCTTCTGTTTCCTTATATATTTTAGACATACTTATGAGCCGTTGTGATCAGTGTCTTCGGCAGATAA
AAGGAGGAATGCCGTCCGGTCGGCAGGCACCGGCTGTTGGTTATGTCTTTTCAAAAACATGAAGGCGGGGA
AATCCAG

P43:
TGATAGGTGGTATGTTTTCGCTTGAACTTTTAAATACAGCCATTGAACATACGGTTGATTTAATAACTGACA
AACATCACCCTCTTGCTAAAGCGGCCAAGGACGCTGCCGCCGGGGCTGTTTGCGTTTTTACCGTGATTTCGT
GTATCATTGGTTTACTTATTTTTTTGCCAAAGCTGTAATGGCTGAAAATTCTTACATTTATTTTACATTTTTA
GAAATGGGCGTGAAAAAAAGCGCGCGATTATGTAAAATATAAAGTGATAGCGGTACCATTATAGGTAAGA
GAGGAATGTACAC

PtrnQ:
GTCGTCTCTTTTTCCCATTTTCCCCAAAAATACAGGGGTTCAAACCATCGTATGTCAGATTGCCAATTAAGA
TGCTTTGTCTATTTAAAAAACGGCCTCTCGAAATAGAGGGTTGTTATTTGAAAGGAATTATCGTATAATTAG
TTGTGCTGACGTTCTCATAACGCAGTCTATAT

Supplementary Figure 1

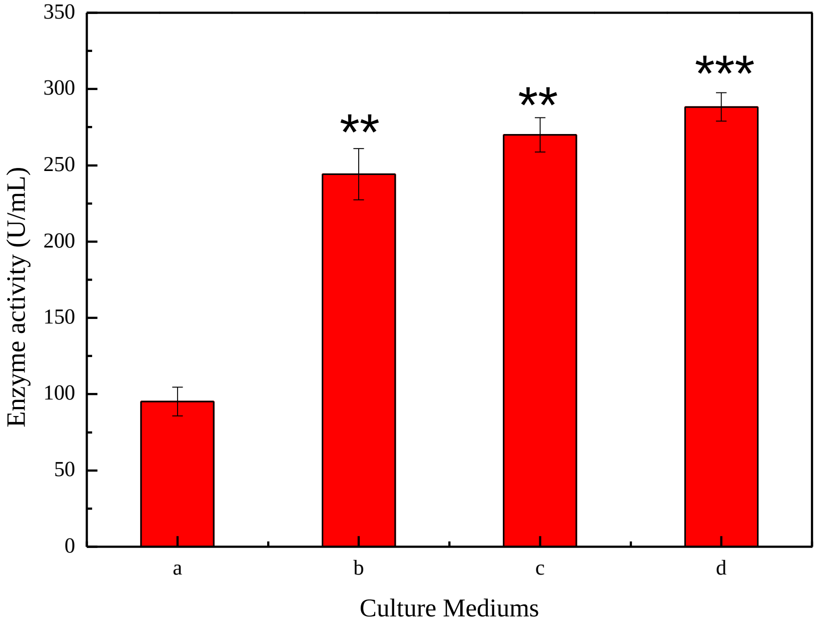

Supplementary Figure 1. The enzyme activity of the strain BAX-9/pHY-P43/BSP-1 in different mediums. a medium with 10 g/L tryptone, 5 g/L yeast extract, 10 g/L NaCl; b medium with 20 g/L tryptone, 10 g/L yeast extract, 10 g/L NaCl; c medium with 30 g/L tryptone, 15 g/L yeast extract, 10 g/L NaCl; d medium with 40 g/L tryptone, 20 g/L yeast extract, 10 g/L NaCl.
